# Carrageenans as biostimulants and bio-elicitors: plant growth and defense responses

**DOI:** 10.1007/s44154-023-00143-9

**Published:** 2024-01-03

**Authors:** Md. Motaher Hossain, Farjana Sultana, Sabia Khan, Jannatun Nayeema, Mahabuba Mostafa, Humayra Ferdus, Lam-Son Phan Tran, Mohammad Golam Mostofa

**Affiliations:** 1 Department of Plant Pathology, Bangabandhu Sheikh Mujibur Rahman Agricultural University, Gazipur, 1706 Bangladesh; 2 College of Agricultural Sciences, International University of Business Agriculture and Technology, Dhaka, 1230 Bangladesh; 3 Department of Agriculture, Faculty of Science, Noakhali Science and Technology University, Noakhali, 3814 Bangladesh; 4 Institute of Genomics for Crop Abiotic Stress Tolerance, Department of Plant and Soil Science, Texas Tech University, Lubbock, TX 79409 USA; 5 Department of Energy Plant Research Laboratory, Michigan State University, East Lansing, MI 48824 USA; 6 Department of Biochemistry and Molecular Biology, Michigan State University, East Lansing, MI 48824 USA

**Keywords:** Antioxidants, Biostimulants, Carrageenan, Defense genes, Metabolites, Oligo carrageenans, Red seaweed, Signaling pathways

## Abstract

In the context of climate change, the need to ensure food security and safety has taken center stage. Chemical fertilizers and pesticides are traditionally used to achieve higher plant productivity and improved plant protection from biotic stresses. However, the widespread use of fertilizers and pesticides has led to significant risks to human health and the environment, which are further compounded by the emissions of greenhouse gases during fertilizer and pesticide production and application, contributing to global warming and climate change. The naturally occurring sulfated linear polysaccharides obtained from edible red seaweeds (Rhodophyta), carrageenans, could offer climate-friendly substitutes for these inputs due to their bi-functional activities. Carrageenans and their derivatives, known as oligo-carrageenans, facilitate plant growth through a multitude of metabolic courses, including chlorophyll metabolism, carbon fixation, photosynthesis, protein synthesis, secondary metabolite generation, and detoxification of reactive oxygen species. In parallel, these compounds suppress pathogens by their direct antimicrobial activities and/or improve plant resilience against pathogens by modulating biochemical changes via salicylate (SA) and/or jasmonate (JA) and ethylene (ET) signaling pathways, resulting in increased production of secondary metabolites, defense-related proteins, and antioxidants. The present review summarizes the usage of carrageenans for increasing plant development and defense responses to pathogenic challenges under climate change. In addition, the current state of knowledge regarding molecular mechanisms and metabolic alterations in plants during carrageenan-stimulated plant growth and plant disease defense responses has been discussed. This evaluation will highlight the potential use of these new biostimulants in increasing agricultural productivity under climate change.

## Introduction

As the world population continues to expand, food security and safety have become one of the most pressing issues to solve. The demand for a growing population necessitates intensive agriculture to ensure higher agricultural productivity. Using chemical fertilizers and pesticides is an indispensable practice in intensive agriculture. Their widespread use hastens the depletion of other essential and minor nutrients and disrupts microbial activity, leading to nutritional imbalance and poor soil fertility (Halim et al. 2004; Singh and Jajpura 2016; Hossain et al. 2017). Many of these toxic farm chemicals pose acute risks to human health and the environment. Furthermore, the production and use of fertilizers and pesticides result in the emission of greenhouse gases, which in turn, contribute to global warming and climate change. Given the escalating negative implications of synthetic chemical-based contemporary agriculture, there is an urge to foster sustainable farming without deteriorating environment. The FAO defines sustainable agriculture as conserving land, water, and genetic resources while being commercially viable, socially acceptable, and environmentally safe (Singh and Jajpura 2016). Sustainable agriculture is based on using good agricultural practices that enhance crop productivity and minimize yield losses from pests and diseases without or with reduced chemical fertilizer or pesticide use (Singh and Jajpura 2016; Hossain et al. 2017). Biotic elicitors can play a very significant role in sustainable agriculture. Various metabolites of plant, microbial and algal origins can act as elicitors that stimulate plant growth, protect plants from diseases and render plant tolerance to abiotic stresses. Proteins, peptides, lipids, polysaccharides and oligosaccharides derived from the cell wall, culture filtrate, and cytoplasm of these organisms are among the potent elicitors documented in various studies (Halim et al. 2004; Singh and Jajpura 2016; Hossain et al. 2017; Hossain and Sultana 2020). These bio-elicitors are usually nontoxic to the environment and lowest energy-intensive yet maintain crop productivity and farm profitability.

Marine organisms are a profound source of valuable metabolites/compounds (Rocha de Souza et al. 2007). Many of these compounds are necessary to survive in a hostile, diverse, and competitive environment (Vo and Kim 2010). In red algae or seaweed, diverse survival tactics due to the synergistic action of many metabolites, such as phycobilins, proteins, polysaccharides, and stored carbohydrates, have evolved in response to these various environmental variables (Wiencke et al. 2006). The anionic sulfated linear polysaccharide carrageenans can protect and strengthen the cell wall against herbivory and wave assaults (Weykam et al. 1997). Carrageenans have a broad range of commercial interests and have been utilized for industrial applications and agriculture (Bebianno et al. 2021). The food and dairy industries and pharmaceutical businesses are a few examples of the many commercial uses for carrageenans (Li et al. 2014; Azevedo et al. 2023). Carrageenans possess important pharmacologically relevant properties (Campo et al. 2009; Ahmadi et al. 2015), as well as they are known to possess antioxidant and antistress potentials, including the ability to scavenge reactive oxygen species (ROS) (Sun et al. 2015).

In recent years, carrageenans have been reported to have important implications in agricultural applications. When applied to various plant species, carrageenans can increase plant growth and a defensive reaction against infections (Bi et al. 2011; Ghannam et al. 2013). As naturally occurring polysaccharides, carrageenans benefit plants without incurring environmental and climate penalties. Hence, utilizing carrageenan to safeguard plant growth and elicit plant defense against biotic stresses is a promising strategy. This review has highlighted recent advancements in the phyco-stimulating and protective roles of carrageenans as bio-stimulants and bio-elicitors, contributing to heightened defense responses and increased plant productivity amid the challenges of climate change. Furthermore, we have explored how the application of carrageenan can enhance plant defense mechanisms and influence biochemical and metabolic processes to bolster resistance against the damage caused by diverse pathogens.

## Origin, composition, physicochemical properties, and industrial applications of carrageenans

Rhodophyta species, such as *Hypnea, Gigartina, Eucheuma*, *Kappaphycus* and *Chondrus crispus,* are the predominant sources of carrageenans (Campo et al. 2009). Carrageenans are typically separated into six primary categories: Kappa (κ), Iota (ι), Lambda (λ), Nu (υ), Mu (μ), and Theta (θ). However, Kappa (κ), Iota (ι), Lambda (λ) are the primary forms used for various purposes. Each of the carrageenans is isolated from a number of seaweed sources. ι-carrageenan, κ-carrageenan, and λ-carrageenan are mainly extracted from *Eucheuma denticulatum*, *Kappaphycus alvarezii*, and various *Gigartina* and *Chondrus* species, respectively (Rudolph 2000). Carrageenans are a type of hydrophilic linear sulfated galactan, which is composed primarily of alternating 3-linked b-D-galactopyranose (G-units) and 4-linked a-D-galactopyranose (D-units) or 4-linked 3,6-anhydro-a-D-galactopyranose (DA-units) (Campo et al. 2009). k-carrageenan comprises D-galactose connected to anhydro-galactose at the C4 position (Vera et al. 2011). λ-carrageenan is formed by two D-galactose, one with a sulfate group at the C2 place and another with sulfates at the C2 and C6 sites. C4-sulfated D-galactose and C2-sulfated anhydro-D-galactose are the building blocks of ι-carrageenan (Campo et al. 2009). Sulfate concentration in carrageenans varies, ranging from 22% in k-carrageenan, 32% (w/w) in ι-carrageenan, and 38% (w/w) in λ-carrageenan (De Ruiter and Rudolph 1997). Since λ-carrageenan has the highest level of sulfation among carrageenans, it shows a more vigorous eliciting activity in plants and other organisms (Mercier et al. 2001; Cunha and Grenha 2016). Several carrageenans have additional substituents, such as modest amounts of terminal xylose, although their precise location is unknown (Van De Velde et al. 2001).

Carrageenans are cellular components characterized by their large molecular weight. The molecular weight of carrageenans can vary depending on several factors, including the specific type of carrageenan, the source seaweed species, and the extraction or processing methods employed. Typically, native seaweed carrageenans have an average molecular weight within the range of 100 to 1000 kDa (Campo et al. 2009), while commercial carrageenan exhibits molecular weight from 30 kDa to 5000 kDa, with an average weight between 200 and 800 kDa (Younes et al. 2018). In contrast, the molecular weight of oligo-carrageenans ranges from a few hundred to several thousand Daltons. Recent studies have demonstrated that reducing the molecular weight of carrageenan can enhance its potential for various biological activities, including its capacity to promote plant growth (Gil 2018; San et al. 2020). Consequently, there is a significant interest in the production of oligo-carrageenans with reduced molecular weights for use in plant-related applications (Castro et al. 2011). Controlled degradation or depolymerization of native carrageenans can result in the formation of smaller oligo-carrageenan molecules (San et al. 2020).

Carrageenan fractions are all water soluble, but insoluble in organic solvents, oil, or lipids. The presence of sulfate groups and their linked cations, such as magnesium, calcium, sodium, and potassium, dominates their water solubility (Pardonche et al. 1985), and the balance between them determines the viscosity of solutions or the strength of carrageenan gels. These unique properties make carrageenans valuable as stabilizing, thickening, and gelling agents in the pharmaceutical and food sectors (Campo et al. 2009).

**Fig. 1:**
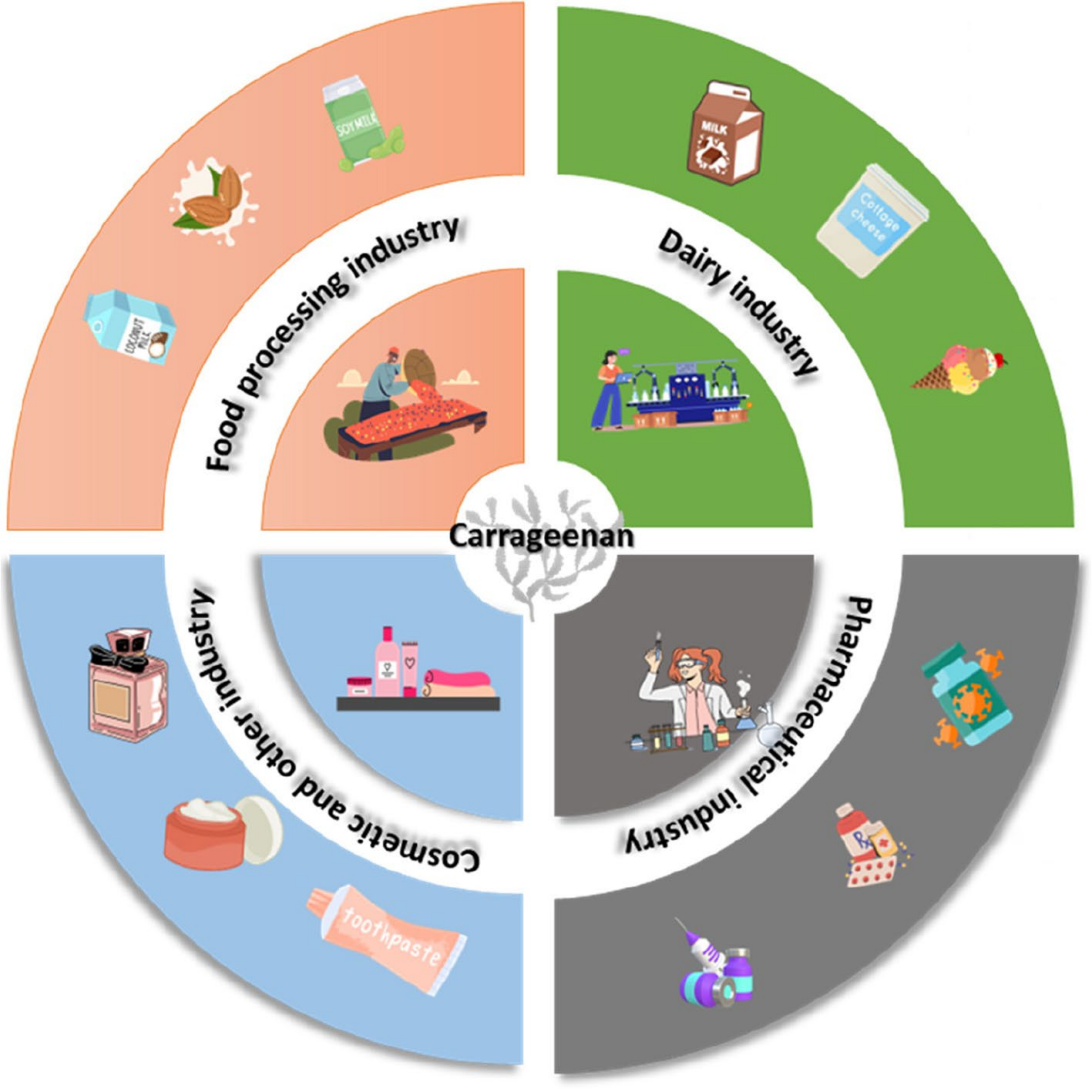
Pictorial representation of the commercial application of seaweed carrageenans in different sectors

Carrageenans have wide-ranging industrial use in diverse sectors, such as food, feed, pharmaceuticals, cosmetics, and other industries (Fig. 1). They contribute to syneresis control, bodying, binding, emulsion stabilization, and dispersal, with a prominent role in the food industry, particularly in dairy products (Stanley 1987). Carrageenans excel at mixing cocoa in chocolate milk, surpassing the capabilities of other gums. When used in dessert gels, ι-carrageenan produces gels with textures that are extremely comparable to those formed by gelatin gels, but with a higher melting point, making them ideal for tropical climates or areas without refrigeration (Azevedo et al. 2023). Additionally, iota gels maintain a soft texture over time, a key attribute for European-style ready-to-eat desserts. Carrageenans also serve as binders in toothpaste and are used in pet diets and air freshener gels (Stanley 1987). Carrageenans have long been studied for their potential health benefits, including anti-inflammatory, anticancer, immunomodulatory, antihyperlipidemic, and anticoagulant effects (Morris 2003; Panlasigui et al. 2003; Zhou and Yu 2004). These natural compounds have demonstrated antiviral properties, inhibiting hepatitis A, herpes virus, and genital human papillomaviruses (HPVs) (Girond et al. 1991; Carlucci et al. 1999; Roberts et al. 2007). While carrageenans were tested as a microbicide for HIV prevention, they proved safe for human usage, but ineffective at the tested concentration. Carrageenans were only effective against HIV at about 1000 times greater concentrations than those needed to suppress HPV (Buck et al. 2006). This suggests that carrageenans could serve as templates for future anti-HIV drug development through chemical modifications, highlighting their ongoing relevance in both industrial and health-related contexts.

## Purification methods and cost of carrageenans

Modern carrageenan extraction techniques aim for high yields while minimizing time and costs (Ortiz-Tena et al. 2017). Two primary methods are used for carrageenan extraction, leading to the production of two grades: semi-refined (SRC) and refined (RC) carrageenans. Each of these methods has distinct principle. For RC extraction, seaweed is cleaned and washed at 95-110 °C with hot alkaline solutions, typically sodium, calcium, or potassium hydroxide (Jönsson et al. 2020). This process creates an alkaline environment, removes the seaweed matrix, and concentrates carrageenan. The “gel press process” using potassium chloride or isopropanol can precipitate carrageenan as needed (Lipnizki 2010). Alcohol purification works for all carrageenans, but gel extraction is effective only for κ-carrageenan (FAO 2014). Squeezing or freeze-thawing removes remaining gel water. After precipitation, carrageenans are dried, ground, and redissolved to obtain a pure solution. In contrast, SRC extraction is a simpler process that doesn’t involve seaweed matrix removal. It relies on aqueous potassium hydroxide to dissolve and remove seaweed non-carrageenan salts, carbohydrates, and proteins in 2 hours at 75 °C (Heriyanto et al. 2018). A gel is formed using potassium reagent and seaweed carrageenan, preventing melting in a hot solution. Soluble proteins, carbs, and salts are removed by washing the residue repeatedly. Alkali-treated seaweed is sliced and crushed into SRC or seaweed flour after 2 days of hot drying (Bono et al. 2014).

Several other carrageenan extraction methods have also been implemented, including enzyme-based, microwave-assisted and ultra sound assisted extraction. Enzyme-based extraction, although promising, has seen limited industrial-scale use. Typically, cellulase, a widely used enzyme, is mixed with ground seaweed and distilled water, followed by boiling for 1 hour at 50 °C. After centrifugation, the supernatants are mixed with 2-propanol, and the resulting precipitated fraction is dried using a freeze dryer (Varadarajan et al. 2009). Microwave-assisted extraction is another efficient method that offers faster drying of high-water-content biomass compared to sun drying and serves as an alternative to solvent-assisted extraction (Sari et al. 2020). This technique involves alkaline treatment followed by microwave extraction at 2450 MHz and full power (Vázquez-Delfín et al. 2014). Initially, 1 g of materials is hydrated in 50 ml of either distilled water (for aqueous extraction) or 3% potassium hydroxide (for alkaline extraction) for 12 hours. Then, a closed-vessel system is used to minimize solvent and analyte loss, accelerating the mass transfer of carrageenan compounds from seaweed samples (Bouanati et al. 2020). Ultrasound-assisted extraction ppears to be simple and is becoming popular (Tiwari and Troy 2015). In this method, dried seaweed is incubated overnight in 80% ethanol at room temperature as pretreatment and then filted before being ultrasounded at 150 W for 15 minutes. Ultrasonic waves convert into mechanical energy rupture the cell wall, reducing particle size and releasing carrageenan (Nigam et al. 2022). After removing algal residues by hot filtering, the filter is left at 4 °C for several hours to produce carrageenan extract as a gel, which was frozen and lyophilized to make powder (Youssouf et al. 2017).

A cost analysis reveals that SRC extraction is more cost-effective than RC extraction because it eliminates expenses related to carrageenan precipitation and solvent recovery. However, SRC results in a lower-quality product often called “seaweed flour” or “alkali-modified flour,” which is not suitable for human consumption due to its coloration and high bacterial population, primarily intended for use in pet food production (Rhein-Knudsen et al. 2015). In contrast, RC extraction yields a higher-quality product known as “raw carrageenan” (Ortiz-Tena et al. 2017). However, enzyme and ultrasound-assisted extraction methods are more environmentally friendly than traditional approaches because they use fewer solvents and less energy (Torres et al. 2021). They also require less extraction time compared to traditional methods. Despite these advantages, the high cost of the enzyme cellulase limits the widespread use of enzyme-assisted extraction on a commercial scale. Ultrasound-assisted extraction, on the other hand, is cost-efficient as it requires minimal equipment and procedures (Tiwari and Troy 2015). Furthermore, ultrasound technology is simpler, faster, and less dependent on the biochemical composition of algae compared to enzyme-assisted extraction (Hahn et al. 2012; Zhu et al. 2017).

## Biological actions of carrageenans in plants

**Fig. 2:**
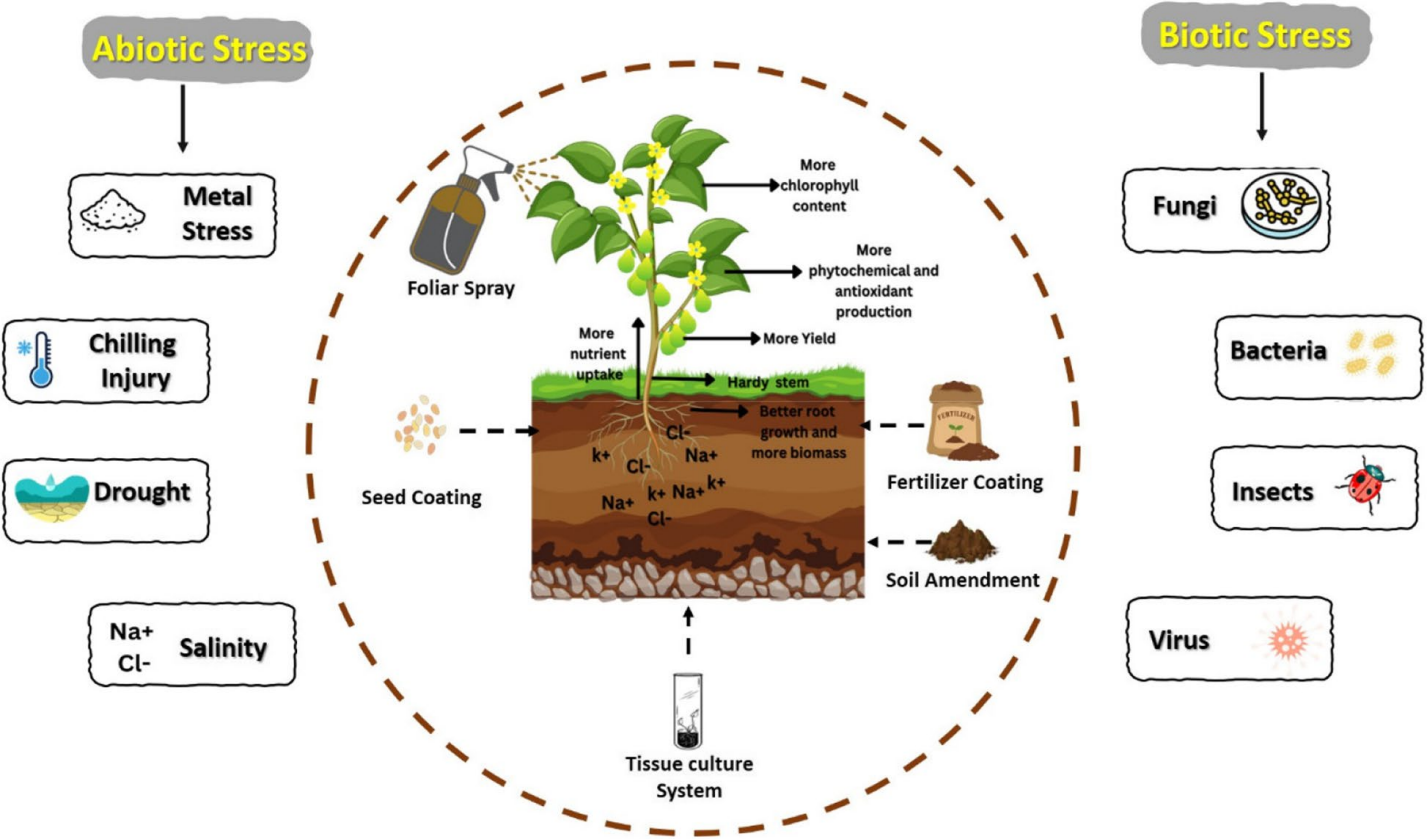
Role of carrageenans in stimulating plant growth and eliciting defense responses against biotic and abiotic stresses. Various application carrageenans trigger various biochemical and metabolic processes, improving plant growth, nutrient uptake, photosynthesis, secondary metabolite contents, antioxidant activity, and defense actions against biotic and abiotic stresses

Seaweed carrageenans have emerged as a promising natural solution to boost plant. In numerous studies, these carrageenans, when applied as foliar spray, soil amendments, seed coating, fertilizer granule coatings or carrageenan solutions in tissue culture media, exhibit multifaceted benefits (Ichi et al. 1986*;* Bi et al. 2011*;* Abad et al. 2016*;* *Abad et al.* 2018a*,* 2018b*;* *Santamaría Vanegas et al.* 2019*;* Arum 2023). They act as biostimulants, enhancing crop emergence, increasing photosynthetic activity, improving nutrient uptake, promoting root development, and ultimately leading to improved yield and quality (Bi et al. 2008*;* Naeem et al. 2012*;* Hashmi et al. 2012) (Fig. 2). By interacting with plants, carrageenans effectively prime plants to withstand various environmental challenges, such as diseases, pests, and abiotic stresses (*Arum* 2023*;* *Ghanbari et al.* 2023*;* *Mola Ali Abasiyan et al.* 2019*;* *Alam* 2018). By utilizing the potential of seaweed carrageenans, we may be able to revolutionize agricultural practices, thereby nurturing greater crop resilience and productivity.

### Effect of carrageenans on plant growth and development

Carrageenans and their oligomeric forms, oligo-carrageenans, have recently been reported to support plant growth in many plant species (Table 1). As a result of their treatment effects, plants grow more rapidly and robustly. *Hypnea musciformis* is a prolific provider of phyco-stimulant carrageenans. Applying *H. musciformis* derived κ-carrageenan around the sowing seeds and on the foliages substantially improved the growth characteristics of chickpea (*Cicer arietinum*), including plant height, pod number, branch number, leaf number, and earlier flowering. Several attributes of maize plants, including plant height, stem diameter, and leaf number, were also significantly influenced by both treatments (Bi et al. 2011). Shoot height and leaf biomass of tobacco plants was increased by applying 1 mg mL^−1^ of κ, λ, and ɩ-carrageenans (Vera et al. 2011). Foliar treatment of κ-carrageenan also increased the shoot length and leaf area of sweet basil (*Ocimum basilicum* L.) while alleviating the unfavorable influence of *Cuscuta campestris* on plant growth (Mousavi et al. 2017). These findings recommended that the k-carrageenan elicitor could be employed as an effective plant growth stimulator for a number of plant species. Seaweed carrageenans applied as liquid fertilizers enhanced rice seed germination, growth, yield, and nutritional quality while also assisting in the establishment of the abundance of beneficial microorganisms in the soils (Tahar et al. 2021). In another study, carrageenans extracted from *K. alvarezii* carrageenan improved rice plant growth, as measured by the growth rate, increased leaf area index, plant height, total tillers, dry matter accumulation, net assimilation rate, and benefit and net return, as well as increased straw and grain yield (Table 1) (van de Tol Castro et al. 2023).

**Table 1:** Effects of different types of carrageenans on growth and yield of various plants

Carrageenan type	Source	Host plant	Effects on plant growth	Refer-ences
λ-carrageenans	Commercially available (Sigma, Chimie, France)	*Brasssica oleracea*	Enhanced the induction of microspore embryogenesis	Lemonnier-Le Penhuizic et al. (2001)
κ-carrageenan	*Hypnea musciformis*	*Cicer arietinum, Zea mays*	Increased plant height, pod number, branches, leaves, early flowering, and induced secondary metabolite contents in chickpea Increased plant height, stem diameter, leaf number, and induced secondary metabolite contents in maize	Bi et al. (2011)
κ, λ, and ɩ-carrageenans	*Ascophyllum nodosum, Rubus fruticosus*	*Nicotiana tabacum*	Increased shoot height and leaf biomass	Vera et al. (2011)
oligo-carrageenans κ, λ, and ɩ	Commercial (Gelymar S.A.)	*N. tabacum*	Increased leaf biomass and plant height	Castro et al. (2011)
γ-irradiated oligo- κ-carrageenan	Commercial (Sigma–Aldrich, USA)	*Foeniculum vulgare*	Increased growth and essential oils contents	Hashmi et al. (2012)
Oligo-carrageenans κ, λ, and ɩ	Commercial (Gelymar S.A., Santiago, Chile)	*Eucalyptus globulus*	Increased plant height, trunk diameter, and holo-cellulose, α-cellulose, and essential oil contents	González et al. (2013b)
κ-carrageenan	Commercial (Sigma Aldrich, USA)	*Catharanthus roseus*	Stimulated seed germination, root growth, shoot elongation, and flower production	Naeem et al. (2015)
Oligo-carrageenans κ	Commercial (Gelymar S.A., Santiago, Chile)	*Pinus radiata*	Improved plant growth	Saucedo et al. (2015)
κ-carrageenan	Commercial (Sigma-Aldrich, U.S.A.)	*Ocimum basilicum*	Increased shoot length and leaf area	Mousavi et al. (2018)
κ-carrageenan	Irradiated by own	*Oryza sativa*	Promoted root growth, tiller number, and development of sturdy and lodging tolerant stems	Abad et al. (2018a)
κ-carrageenan	Commercial *(*Shemberg Corporation, Philippines)	*Arachis hypogaea*	Improved seed germination, plant tallness, flowering, pod number, seed weight, and yield	Abad et al. (2018b)
κ-carrageenan	*Kappaphycus alvarezii*	*Zea mays*	Promoted growth and grain yield	San et al. (2020)
λ-carrageenan	Commercial (Sigma Aldrich, Germany)	*Musa acuminata*	Increased root length, plant length, pseudo stem diameter, and fresh weight	Thye et al. (2022)
Carrageenans	*Kappaphycus alvarezii*	*Oryza sativa*	Improved rice plant growth, increased leaf area index, plant height, total tillers, growth rate, dry matter, net assimilation rate, straw and grain yield	van de Tol Castro et al. (2023)

**Fig. 3:**
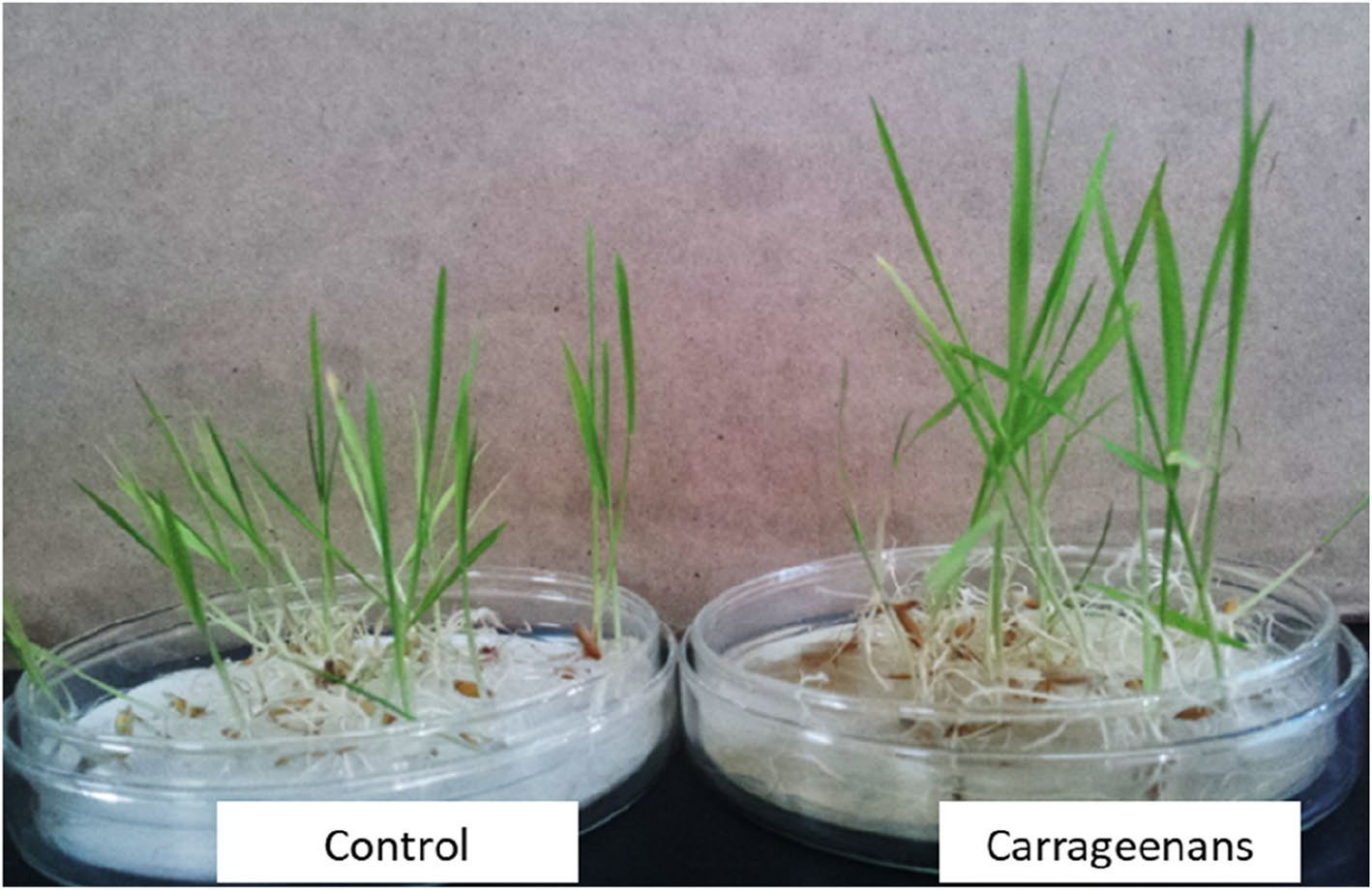
Effect of carrageenans derived from *Hypnea musciformis* on the growth of rice seedlings. Carrageenans were added to Petri dishes, while water was added control plants. Seedling growth was observed two weeks after sowing

Carrageenans extracted from *H. musciformis* have also been shown to enhance seedling height and biomass production in rice (Fig. 3). The chemical constituent of *H. musciformis* carrageenan has widely been reported as k-carrageenan (Cosenza et al. 2014). Recently, the *H. musciformis* κ- carrageenan was observed to have anti-inflammatory activity and no in vivo toxicity (Brito et al. 2019). λ-carrageenan has the highest sulfate content and is expected to show increased eliciting activity. However, few investigations have been undertaken to determine its effect on plant growth. Coupled with heat stress, adding λ-carrageenan significantly increased the final yields of microspore-derived embryos of *Brassica oleracea var. italica*, by as much as twofold in the most reactive treatment (Lemonnier-Le Penhuizic et al. 2001). The carrageenan oligomers exhibited the most significant microspore induction effects, while treatment for only 30 minutes was sufficient to accelerate embryogenesis, and the two optimum doses were 170 nM and 34 μM. Hence, this study enlightens the potential of λ-carrageenan as an innovative promoter in the production of doubled-haploid plants from microspore cultures. A recent study has examined the influences of λ-carrageenan on the growth of banana (*Musa acuminata* cv. Berangan) plants. Plant height, pseudo-stem diameter, root length, root structure, and fresh biomass weight were substantially enhanced in banana plants sprayed with λ-carrageenan at a concentration of 750 ppm (Thye et al. 2022), suggesting that λ-carrageenan can enhance plant growth at optimum concentration.

Several investigations have found that carrageenans do not stimulate plant growth or have more potent effects until they are depolymerized into oligo carrageenans (Thye et al. 2022). According to Gil (2018), lowering the molecular weight of carrageenan increases its efficiency as a biostimulant in plants. Depolymerization or degradation of carrageenans is done through various processes, including acid hydrolysis, γ-irradiation, ultrasonic treatment, and microbial enzymolysis. These processes induce the splitting of the glycosidic bonds generating low molecular weight oligomers. Oligomers generated from depolymerized carrageenan may also confer the plant growth-stimulating action of carrageenans. In an earlier study examining oligo-carrageenans’ effects on plant growth, oligo-carrageenan κ produced through γ-irradiation of carrageenans improved the growth of rice roots and shoots (Abad et al. 2004). Tobacco plants responded positively, showing increased height and leaf biomass to the treatment with oligo-carrageenans κ, λ, and ɩ produced through acid hydrolysis, particularly oligo-carrageenan κ was the most effective among them (Moenne 2010). In another study, when tobacco plants var. Burley were sprayed with oligo-carrageenans κ, λ, and ɩ at a dose of 1 mg mL^−1^, the leaf biomass was augmented significantly (van de Tol Castro et al. 2023). No growth stimulation occurred in tobacco plants unless κ-, λ-, and ɩ-carrageenans had not been depolymerized. In the medicinal plant *Foeniculum vulgare*, the γ-irradiated oligo-carrageenan promoted the growth and fenchone, essential oils, and trans-anethole content, with the most effective concentration being 80 mg L^−1^ (Hashmi et al. 2012). The unirradiated carrageenan did not affect the growth traits of *F. vulgare*. Likewise, *Eucalyptus globulus* sprayed with acid-hydrolyzed oligo-carrageenans κ, λ, and ɩ exhibited an improvement in trunk diameter and height as well as in the contents of holo-cellulose, α-cellulose, and essential oils (González et al. 2013b). The most promising of the three oligo-carrageenans was oligo-carrageenan κ. Oligo-carrageenan κ also enhanced the growth of *Pinus radiata* (Saucedo et al. 2015) and *Catharanthus roseus* (Naeem et al. 2015). However, the duration of the induction effect of oligo-carrageenan κ on these two trees differs. The phyco-stimulating influences of oligo-carrageenan κ persisted in *Eucalyptus* plants for years, while this effect was only temporary in pine trees (Saucedo et al. 2015). Oligo carrageenan κ is also an effective bio-stimulator for several field and horticultural plants, especially in the case of maize, rice, chrysanthemum, potato, peachy, fennel, mung bean, mustard, and peanuts (San et al. 2020).

The formulation development is crucial for the commercial application of carrageenans and oligo-carrageenans, which have been demonstrated to positively impact plant growth and development in many studies. On rice, the effectiveness of foliar spraying with several formulations of the γ-irradiated oligo-carrageenan solution was researched and evaluated in the field (Abad et al. 2018a). The best formulation was mass-produced for field use. Multilocation trials of over 1600 ha of rice fields in various locations of the Philippines revealed an average production increase of around 20%. The rice plants grew deep roots, more tillers, and robust, lodging-resistant stems, making plants more resistant to typhoons (Abad et al. 2018a). Agricultural experts at the University of the Philippines’ National Crop Protection Center in Los Baos examined the effect of carrageenan as a plant growth stimulator on over 5000 ha of rice plants (Gil 2018). Researchers observed that sprayed regions produced crops with 65% higher yields than the control plants while using only half the prescribed fertilizer amount in a trial in Pulilan, a central province of Bulacan. The fertilizing effect of carrageenan was long-lasting, and the grain-bearing apex of the stem was brimming with grains (Gil 2018). Therefore, using carrageenans and oligo carrageenans as plant growth stimulants is the solution to shorten the harvesting time. Indeed, the carrageenan-based technique can boost crop yields, which in turn can improve farmers’ livelihoods.

### Effect of carrageenans on plant defenses against diseases

Phytopathogens cause substantial yield losses in economically and agriculturally important crops. Over the past few years, great emphasis has been placed on the quest for new classes of antipathogenic compounds that are safe for the environment but most successful in attaining crop protection (Mercier et al. 2001; Vera et al. 2011). Furthermore, there has always been a limited option for directly controlling viruses using chemical techniques. Since viruses rely on plant cell machinery to proliferate, annihilating them with toxic chemicals is problematic. Seaweed carrageenans have emerged as good candidates for controlling viruses, fungi, and bacteria in different host plants (Table 2). Tobacco mosaic virus (TMV) is the most stable plant virus, capable of infecting dozens of plant species. Several studies have shown that carrageenans from several red marine algae may provide a natural way to trigger induced responses that show enhanced resistance against TMV. The effect of κ/β-carrageenan derived from *Tichocarpus crinitus* on foliar infection of TMV in Xanthinc tobacco was investigated. When κ/β-carrageenan was applied to leaves, the number of necrotic lesions reduced by 87% compared with the leaves treated with TMV alone (Nagorskaya et al. 2010). Similar κ/β-carrageenan treatment induced a number of lytic events in *Datura stramonium* plants that prevent the accumulation and transport of Potato Virus X particles within the cell (Nagorskaya et al. 2008).

**Table 2:** Effects of carrageenans on plant defense against various plant pathogens

Carrageenan type	Source	Host plant	Pathogen	Effect	References
κ/β-carrageenan	*Tichocarpus crinitus*	*Nicotiana tabacum*	*Tobacco mosaic virus (TMV)*	Decreased necrotic lesion number by 87% in leaves.	Nagorskaya et al. (2010)
κ/β-carrageenan	*Tichocarpus crinitus*	*Datura stramonium*	*Potato Virus X*	Prevented accumulation and transport of Virus particles within the cell.	Nagorskaya et al. (2008)
λ-carrageenan	*Acanthophora spicifera*	*Arabidopsis thaliana*	*Sclerotinia sclerotiorum*	Reduced infection	Sangha et al. (2010)
Oligo-carrageenans κ, λ and ɩ	Commercial (Gelymar S.A., Santiago, Chile)	*N. tabacum*	*TMV, Botrytis cinerea, Pectobacterium carotovorum*	Complete to partial suppression of infections at local and systemic levels	Vera et al. (2012)
κ-carrageenan	*Hypnea musciformis*	*Nicotiana tabacum*	*TMV*	Reduced TMV infection	Ghannam et al. (2013)
λ-carrageenan	*Acanthophora spicifera*	*Solanum lycopersicum*	Tomato Chlorotic Dwarf Virus (TCDVd)	Exhibited increased resistance to TCDVd	Sangha et al. (2015)
κ-carrageenan	Commercial (Sigma-Aldrich, U.S.A.)	*Ocimum basilicum*	*Cuscuta campestris*	Decreased infestation by about 26%	Mousavi et al. (2018)
κ-carrageenan	*Kappaphycus alvarezii*	*Capsicum annuum*	*Colletotrichum gloeosporioides*	Inhibited growth	Mani and Nagarathnam (2018)
Modified κ-carrageenan	*Kappaphycus alvarezii*	*Oryza sativa*	*Xanthomonas oryzae pv. oryzae*	Shorter bacterial leaf blight lesions	Bayot et al. (2018)
Carrageenan	–	*O. sativa, Vigna radiata*	*Cercospora leaf spot and rust*	Lowest leaf spot and rust incidences	Gatan and Gatan (2019)
λ-carrageenan	*Acanthophora spicifera*	*Hevea brasiliensis*	*Phytophthora palmivora*	Reduced infection	Pettongkhao et al. (2019)
λ-carrageenan	Sigma	*Triticum aestivum*	*Zymoseptoria tritici*	Developed fewer disease symptoms	Le Mire et al. (2019)
κ-carrageenan	*Kappaphycus alvarezii*	*Carica papaya*	*Papaya ringspot virus* (PRSV)	Showed complete inhibition	Premchand et al. (2021)
Algomel PUSH®	*Solieria chordalis*	*T. aestivum*	Powdery mildew (*Blumeria graminis*)	Suppressed disease for a period of 20 days	Dal Bosco Ducatti et al. (2022)

TMV infection was reduced in tobacco plants, when treated with *H. musciformis* sulfated polysaccharide 4 (SPS4) containing 98% κ-carrageenan (Ghannam et al. 2013). Tomato plants treated with carrageenans have been demonstrated to resist viroids of Pospiviroidae family, such as the Tomato Chlorotic Dwarf Virus (TCDVd) (Sangha et al. 2015). TCDVd is a destructive Pospiviroid that infects many solanaceous vegetables and ornamental plants, including tomatoes. Tomato plants administered with λ-carrageenan exhibited increased resistance to TCDVd (Sangha et al. 2015). Tomato plants exposed to water had a greater abundance of TCDVd transcripts than those exposed to λ-carrageenan. This finding suggests that λ-carrageenans reduce TCDVd in tomato plants, most likely via regulating viroid replication (Sangha et al. 2015). In various regions of the Philippines, rice plants treated with foliar sprays of γ -irradiated carrageenan solutions displayed enhanced resistance to the tungro virus (Abad et al. 2018a). Therefore, carrageenan has been found to have a plant-elicitor effect with tungro virus resistance.

Carrageenans are highly effective against pathogenic fungi. The destructive necrotrophic pathogen *Sclerotinia sclerotiorum* can infect a wide variety of hosts (Hossain et al. 2023)*.* Genetic resistance against the fungus is infrequent (Prova et al. 2018; Islam et al. 2021; Jahan et al. 2022). However, *Arabidopsis thaliana* showed enhanced resistance to *S. sclerotiorum* infection after being treated with λ-carrageenan (Sangha et al. 2010). Chili is vulnerable to anthracnose disease caused by the fungus *Colletotrichum gloeosporioides,* resulting in severe yield damage (Than et al. 2008). Attempts are being rendered worldwide to control the disease using various techniques. Adding κ-carrageenan from *K. alvarezii* in the growth media led to a dose-dependent suppression of *C. gloeosporioides* growth (Mani and Nagarathnam 2018). This shows that κ-carrageenan can be utilized as a strong elicitor of anthracnose resistance in chili plants. *Septoria tritici* blotch, caused by *Zymoseptoria tritici*, is a severe disease in Europe. Each year, up to 40% of the yield is lost due to the infection of wheat (Rudd 2015). λ-carrageenan, has been shown to be a potential elicitor of wheat defenses against this pathogen (Le Mire et al. 2019). Plants treated with λ-carrageenans developed fewer *Z. tritici* disease symptoms than controls. Under semi-controlled conditions, foliar application of λ-carrageenan provided approximately 70% protection against *Z. tritici* (Le Mire et al. 2019). In Brazil, a commercial *Solieria chordalis* carrageenan-rich product, Algomel Push, has been shown to be an effective elicitor for suppressing foliar diseases, particularly the powdery mildew (*Blumeria graminis*) of wheat for a time span of roughly 20 days (Dal Bosco Ducatti et al. 2022).

In the Philippines, field experiments were piloted to formulate integrated crop management (ICM) for mungbean (*Vigna radiata*) by utilizing carrageenans and other biocontrol agents under various cropping systems (Gatan and Gatan 2019). In rice-mungbean cultivation systems, the cultivar Pagasa 19 treated with carrageenan had the lowest Cercospora leaf spot and rust incidences and higher yield than control plants. Under a corn-mungbean cropping system, Pagasa 19 treated with carrageenan had the most significant yield improvement. In other trials, Cercospora leaf spot in carrageenan-treated Pagasa 19 plants was lessened by 25.6%, while yield/ha was increased by 62.3% compared to untreated Pagasa 19. Carrageenan was also effective against Cercospora leaf spot and rust in the cultivar Pagasa 7, giving the highest yield (1.37 tons/ha) (Gatan and Gatan 2019). These results show that an improved ICM system incorporating carrageenan is essential for reducing crop loss, enhancing soil condition, and increasing yield in mungbean.

While much research has been done on bacteria, one key challenge has been the lack of cost-effective chemical control against bacterial diseases in the field. Carrageenans show antibacterial capabilities when employed against specific plant pathogenic bacteria. Bacterial leaf blight (BLB) is a destructive disease of rice that is difficult to control in the field (Bayot et al. 2018). The effectiveness of modified κ-carrageenan (MKC) as an inducer of resistance against rice bacterial leaf blight was assessed (Bayot et al. 2018). Blight lesions were shorter on plants sprayed with a 100 ppm solution of MKC three times, once every 7 days, beginning 30 days after planting. Induced resistance was also demonstrated when the roots of rice plants (Mestizo 20 hybrid) were immersed in a 100 ppm solution of MKC for 5 minutes. Consequently, the treated plants had shorter blight lesions than the untreated plants (Bayot et al. 2018). Carrageenans can have an inhibitory effect on parasitic higher plants like *Cuscuta*. *Cuscuta campestris* is a hollo stem parasite that infects Sweet basil, a valuable commercial plant, and inhibits the growth and development of infected plants. *C. campestris* is notoriously hard to eradicate. To solve this problem, research determined the outcomes of applying κ-carrageenan on sweet basil in preventing invasion and infestation of *C. campestris*. κ-carrageenan treatment enhanced basil branch length and leaf area while decreasing *C. campestris* infestation by around 26% (Mousavi et al. 2018). Furthermore, the efficacy of carrageenans seems broad spectrum. Tobacco plants treated with carrageenans were protected from several phytopathogens (Vera et al. 2012). Spraying oligo-carrageenans κ, λ, and ɩ on tobacco leaves was shown to induce protection against local infection by *Botrytis cinerea,* TMV, and *Peptobacterium carotovorum* (Vera et al. 2012). Treatment with higher oligo-carrageenan concentrations, more frequent treatments, and longer elapsed times improved defenses against TMV and *P. carotovorum* with equivalent efficiency by all three oligo-carrageenans as well as against *B. cinerea*, principally by oligo-carrageenans λ and ɩ. Additionally, oligo-carrageenans caused a total inhibition of *B. cinerea* infection and a partial repression of TMV and *P. carotovorum* infections at the systemic level (Vera et al. 2012). Therefore, carrageenans and oligo-carrageenans can trigger plant defense responses against a wide array of pathogens, allowing them to combat infections better.

## Molecular mechanisms and metabolic changes in plants induced by carrageenans

Carrageenans and oligo-carrageenans influence a wide range of biochemical reactions and pathways in plants. By altering plant physiology and metabolism, carrageenan has been reported to be particularly successful in inducing high levels of primary and secondary metabolites in agricultural plants (Bi et al. 2008). Some metabolites are critical for plant growth and development (Rosenthal 1991), while others protect plants against pathogens (Schäfer and Wink 2009; Zaynab et al. 2018). Furthermore, carrageenans are excellent inducers of plant signaling pathways and gene expression that serve a variety of functions for plants (Mercier et al. 2001; Hashmi et al. 2012).

### Plant growth-promoting mechanisms

Carrageenans and oligo-carrageenans enhance plant growth and development by influencing numerous plant processes (Table 3). Some of the key physiological and metabolic processes that carrageenans affect are photosynthesis and its auxiliary routes, cell division, pyrimidine and purine synthesis, and nitrogen and sulfur (S) assimilation (Shukla et al. 2016). In *B. oleracea*, oligo-carrageenans seem to function as signaling compounds that induce microspore embryogenesis (Lemonnier-Le Penhuizic et al. 2001). Interestingly, as the amount of sulfate substituents in oligo-carrageenans raised, so did their effectiveness in causing microspore embryogenesis. Treatment of chickpea, pea (*Pisum sativum*), carrot (*Daucus carrota*), and potato (*Solanum tuberosum*) tissues with high molecular weight crude elicitor preparations of red algal plant *H. musciformis* induced a high level of secondary metabolites (Bi et al. 2008). Later, the polysaccharide elicitor obtained from red seaweed was identified as k-carrageenan (Bi et al. 2011). The k-carrageenan treatment in chickpea and maize (*Zea mays*) significantly also triggered high levels of induced secondary metabolite contents in different plant parts, impacting the chickpea and maize growth (Bi et al. 2011). Oligo-carrageenans have been described to enhance plant height and leaf biomass in tobacco through elevated chlorophyll content, net photosynthesis through increased PSII activity, nitrogen (N) assimilation, carbon (C) fixation, and ribulose 1, 5 bisphosphate carboxylase/oxygenase (rubisco) activity (Munoz et al. 2011). Rubisco is the primary enzyme involved in carbon fixation. It converts atmospheric CO_2_ into organic carbon via the Calvin-Benson cycle (Yap and Lai 2017), producing glucose necessary for plant growth. According to Vera et al. (Vera et al. 2011), κ, λ, and ɩ-carrageenans improved rubisco activity, net photosynthesis, and glutamate dehydrogenase synthesis, all of which are important for nitrogen absorption, basal metabolism, and cell proliferation. Adding oligo-carrageenans κ, λ, and ɩ boosted tobacco leaf biomass by improving chlorophyll content, net photosynthesis, C and N assimilation, purine and pyrimidine synthesis, fatty acid synthesis, enzyme activity in the Krebs cycle and levels of transcripts encoding cyclins (A and B) and cyclin-dependent protein kinases (CDKs) (van de Tol Castro et al. 2023). Cyclins and CDKs are key to cell cycle control (Fabian et al. 2000). Thus, the increased biomass has been proposed to be the result of the stimulatory effect of oligo-carrageenans-induced rubisco activity, chlorophyll content, secondary metabolites and cell cycle regulatory proteins (González et al. 2013a).

**Table 3:** Mechanisms of plant growth promotion by carrageenans

Carrageenan	Host plants	Mechanisms*	References
κ-carrageenan	Chickpea and Maize	Induced production of secondary metabolites	Bi et al. (2011)
κ, β and ι-oligo-carrageenan	*Nicotiana tabacum*	Increased chlorophyll, net photosynthesis, nitrogen uptake, carbon fixation, and Rubisco activity	Munoz et al. (2011)
κ, β and ι- oligo-carrageenan	*Nicotiana tabacum*	Increased purine, pyrimidine, fatty acid, enzyme activity in the Krebs cycle, and transcripts for cyclins (A and B) and cyclin-dependent protein kinases (A and B)	Castro et al. (2011)
Oligo-carrageenan	*Pinus radiata*	Enhanced C, N, and S assimilation, basal metabolism-related enzymes for NADPH production, IAA and GA3	Saucedo et al. (2015)
Oligo-carrageenans κ, λ, and ɩ	*Eucalyptus globulus*	Increased chlorophyll and photosynthesis	González et al. (2013b)
κ-carrageenan	*Eucalyptus globulus*	Increased NADPH, ASC, and GSH syntheses, TRR/TRX activities, photosynthesis and basal metabolism	González et al. (2014a)
κ-carrageenan	*Eucalyptus globulus*	Stimulated the production IAA, GA3, and trans-zeatin	González et al. (2014b)
λ-carrageenan	*Musa acuminata* cv. Berangan (AAA)	Enhanced chlorophyll, protein and phenolic content, secondary metabolites, CAT, POD and expression of *RBCL*, *CAO*, *SAMS*, *TCM*, *PRX* and *CAT* genes.	Thye et al. (2022)

*NADPH nicotinamide adenine dinucleotide phosphate, *ASC* ascorbate, *GSH* glutathione, *TRR/TRX* thioredoxin reductase/thioredoxin systems, *POD* peroxidase, *CAT* catalase, *ROS* reactive oxygen species, *IAA* indole acetic acid, *GA3* Gibberelic acid, *RBCL* ribulose-1,5-bisphosphate carboxylase, *CAO* chlorophyllide an oxygenase, *SAMS* S-adenosylmethionine synthase, *TCM* trans-cinnamate 4-monooxygenase, *PRX* Class III peroxidase

The processes by which κ-carrageenan facilitated the *Eucalyptus globulus* growth were investigated (González et al. 2014a). The hypothesized mechanisms by which κ-carrageenan promotes *Eucalyptus* growth include its effects on reduced nicotinamide adenine dinucleotide phosphate (NADPH), ascorbate (ASC), and glutathione (GSH) synthesis, thioredoxin reductase activity/thioredoxin systems (TRRs/TRX), and crosstalk between biological processes (González et al. 2014a). Through the TRRs/TRX, NADPH participates in regulating basal metabolism and thus, plant growth (González et al. 2014a; González et al. 2014b). Similarly, treating *Pinus radiata* with oligo-carrageenan-κ resulted in increased chlorophyll contents and higher activity of rubisco, glutamate dehydrogenase (GDH), O-acetylserine(thiol)-lyase (OAS-TL), and ASC (Saucedo et al. 2015). The basal metabolism enzymes are associated with C, N, and S assimilation and amino acid, purine, pyrimidine and nucleotide biosynthesis, resulting in plant growth (Castro et al. 2011; Saucedo et al. 2015).

In addition to its role in N metabolism, GDH is crucial in keeping the cellular supply of carbon and nitrogen in equilibrium (Miflin and Habash 2002). OAS-TL enzymes are very important for S assimilation and catalyze the final reaction in S assimilation (Álvarez et al. 2011). By contributing to the transition of G1 to the S phase of the cell cycle, ASC and GSH play significant roles in cell division (Vernoux et al. 2000). In addition, GSH protects plant cells from oxidative stress (Jiang et al. 2012). Equally, the increased ASC level in oligo-carrageenans-treated plants may protect chloroplast from photo-oxidative stress, inducing C fixation in chloroplasts, cell cycle operation and cell division process resulting in an improvement in plant growth (Saucedo et al. 2015). Thye et al. (Thye et al. 2022) showed that λ-carrageenan stimulates banana plant growth by increasing photosynthesis, protein and secondary metabolites biosynthesis, catalase (CAT) and peroxidase (POD) activity, which agreed with the observed increase in chlorophyll, protein and phenolic content. A rise in protein content may stimulate secondary metabolite biosynthesis, resulting in increased plant growth (Castro et al. 2011; González et al. 2013a). Enhanced expression of ribulose-1,5-bisphosphate carboxylase (*RBCL*), chlorophyllide an oxygenase (*CAO*), S-adenosylmethionine synthase (*SAMS*), trans-cinnamate 4-monooxygenase (*TCM*), Class III peroxidase (*PRX*) and catalase (*CAT*) genes in treated banana plants (Thye et al. 2022) also indicated that λ-carrageenan activated key metabolic processes involved in plant growth. In addition, enhanced POD and CAT activity contributes to dramatically decreasing the harmful effect of H_2_O_2_, preserving cellular homeostasis in banana plants.

**Fig. 4:**
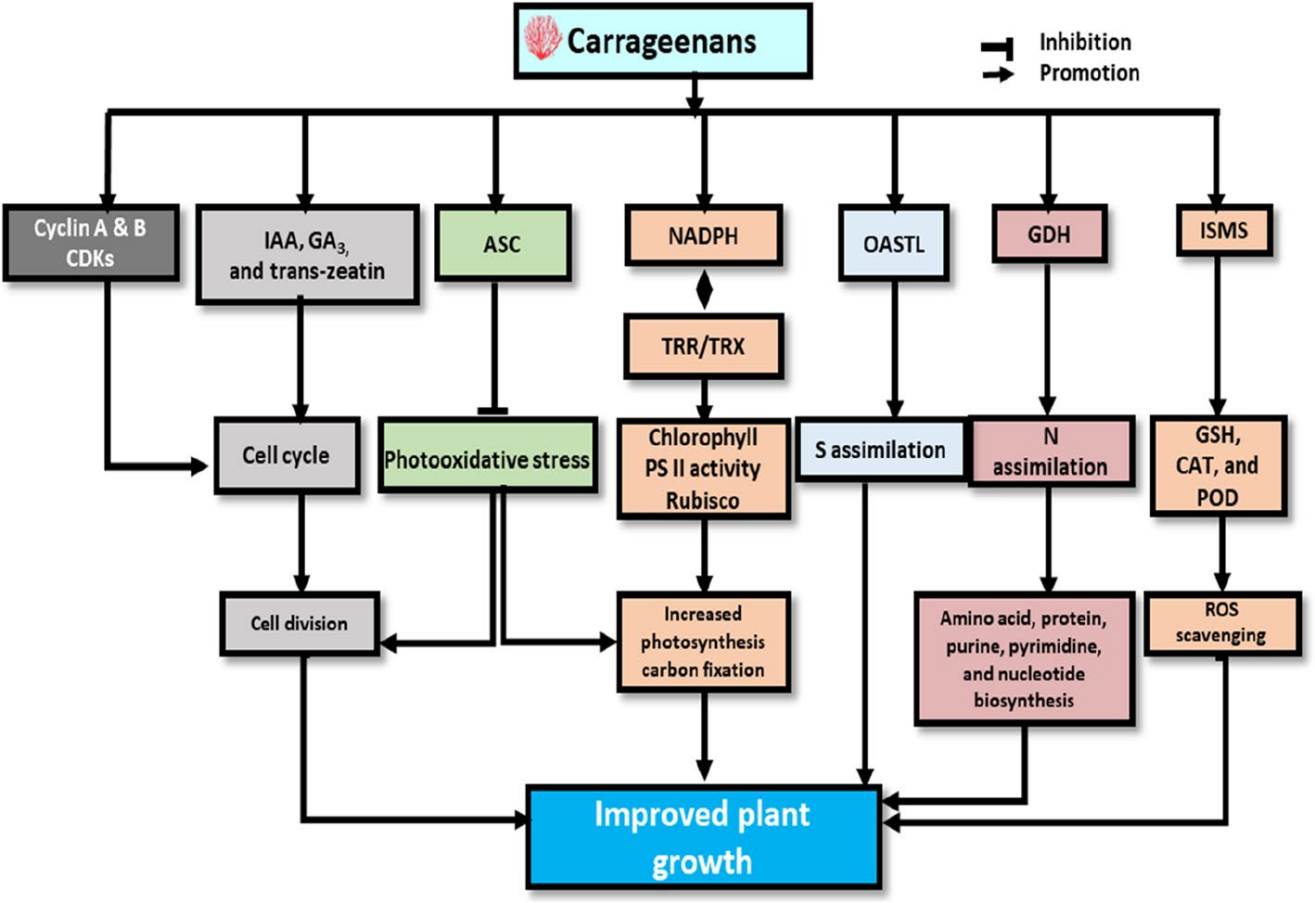
Mechanisms involved in plant growth promotion by carrageenans. Biochemical changes induced by carrageenan application in plants are indicated. CDKs, cyclin-dependent protein kinases; NADPH, nicotinamide adenine dinucleotide phosphate; OASTL, O-acetylserine(thiol)-lyase), GDH, glutamate dehydrogenase; ASC, ascorbate; GSH, glutathione; TRR/TRX, thioredoxin reductase/thioredoxin systems; ISMS, induced secondary metabolites; POD, peroxidase; CAT, catalase; ROS, reactive oxygen species; IAA, indole acetic acid; GA3, Gibberellic acid

Phytohormones are likely to play important roles in regulating the growth of carrageenan-induced plants. According to research by Gonzalez et al. (González et al. 2014b), k-carrageenan has been found to stimulate the production of plant growth hormones, such as indole-3-acetic acid (IAA), gibberellic acid (GA3), and trans-zeatin in *E. globulus*. Similar observations were made by Saucedo et al. (Saucedo et al. 2015), who demonstrated that k-oligo-carrageenan raised IAA and GA3 levels in *Pinus radiata* trees. IAA is the major auxin that controls apical dominance, cell division, tissue elongation, and differentiation, and responses to light and gravity (Zhao 2010). Changes in IAA concentration have the most significant effect on root growth and development. GA3 is known to promote plant height, growth, dry matter accumulation, and yield in various crops (Noor et al. 2017). Zeatin, an adenine-based cytokinin, stimulates plant growth by accelerating cell division and encouraging the formation of lateral buds (Noor et al. 2017). Thus, the application of oligo-carrageenans appears to impact plant growth by affecting the synthesis of NADPH, GSH, and ASC syntheses, the activity of thioredoxin reductase/thioredoxin systems, the metabolic processes required for C, N, and S assimilation, photosynthesis, the synthesis of phytohormones and cell division. The mechanisms of carrageenans on plant growth in various plants are summarized in Fig. 4.

While many of the findings regarding the underlying mechanisms remain at a relatively superficial level, there is a notable scarcity of studies investigating the interaction between plants and carrageenans. These gaps include the identification of target molecules for carrageenan recognition and other related research. There is also a contention that plants may possess the ability to recognize the particular oligomers of natural polysaccharides, which subsequently elicit growth, development, and defence reactions in plants (Darvill et al. 1992). It is also considered that carrageenan oligomers may function in a manner analogous to plant growth regulators, that act as endogenous growth elicitors and function as singnaling molecules to trigger the synthesis of various enzymes and activate different plant responses by effectively modulating gene expression, particularly in the context of plant stress conditions (Ma et al. 2010).

### Eliciting plant defense mechanisms against diseases

Plants treated with carrageenans show well-organized mechanisms to counteract various plant pathogens (Table 4). Several mechanisms are assumed to be implicated in the inhibitory effect of carrageenans on plant viruses. Plants treated with carrageenans prevent virus particle accumulation and translocation within the cell (Nagorskaya et al. 2008). It is observed that carrageenans treatment marginally stimulates lytic processes, resulting in the obliteration of viral particles, and can be regarded as one of the defense mechanisms preventing virus intracellular buildup. Another carrageenan-induced method of antiviral defense in plant cells is the formation of virus-specific laminar structures capable of binding virus particles and, therefore, preventing their intracellular translocation and reproduction (Nagorskaya et al. 2008). TCDVd in tomato plants is most likely controlled by λ-carrageenan through the inhibition of viroid replication (Sangha et al. 2015). Carrageenans may also exhibit direct antifungal activities. For instance, adding κ-carrageenan in the growth media resulted in a dose-dependent inhibition of *Colletotrichum gloeosporioides* (Mani and Nagarathnam 2018). Microscopic studies revealed that κ-carrageenan causes a considerable time-dependent increase in plasma membrane permeability of *C. gloeosporioides* and thus suppresses the pathogen growth.

**Table 4:** Mechanisms of plant defense against diseases by carrageenans

Carrageenan type	Causal organisms	Host plant	Mechanisms involved*	References
λ-carrageenan	*Phytophthora parasitica*	*Nicotiana tabacum*	Enhanced SA content, expression of JA and ET-inducible *LOX* and *ACO*, and transcript levels encoding a chitinase, sesquiterpene cyclase, and proteinase inhibitor	Mercier et al. (2001)
κ/β- carrageenan	*Potato Virus X*	*Datura stramonium*	Stimulated lytic processes	Nagorskaya et al. (2008)
λ-carrageenan	*Sclerotinia sclerotiorum*	*Arabidopsis thaliana*	Triggered JA-dependent mechanism involving the transcription of the *PR3*, *PDF1*.2, and *AOS* defence genes	Sangha et al. (2010)
Oligo-carrageenan	*TMV, Botrytis cinerea, Pectobacterium carotovorum*	*N. tabacum*	Stimulated PAL activity and various phenylpropanoid compounds of JA pathway	Vera et al. (2012)
κ, λ & ι- carrageenan	*Trichoplusia ni*	*A. thaliana*	Increased expression of JA and SA-responsive genes *PDF1*.2 and *PR1*	Sangha et al. (2010)
κ-carrageenan	*TMV*	*N. tabacum*	Increased expression of SA-dependent genes *PR1a*, *PR2*, and *PR5* and JA-regulated genes *PR3* and *Def1*.2	Ghannam et al. (2013)
λ-carrageenan	*TCDVd*	*Solanum lycopersicum*	Inhibited virus replication and elevated expression of JA-dependent proteins LOX, AOS, and PR	Sangha et al. (2015)
	*Cuscuta campestris*	*Ocimum basilicum*	Higher H_2_O_2_ levels and CAT, PAL, and SOD activity but not MDA, other aldehydes, PPO, LOX, or POD.	Mousavi et al. (2018)
κ-carrageenan	*Colletotrichum gloeosporioides*	*Capsicum annuum*	Induced SA-inducible *PR1*, *PR5*, and *NPR1* genes and JA-inducible *PDF1*.2 genes	Mani and Nagarathnam (2018)
λ-carrageenan	*Phytophthora palmivora*	*Hevea brasiliensis*	Boosted SA and scopoletin accumulation and SA-responsive gene expression. Suppress JA-responsive gene	Pettongkhao et al. (2019)
λ-carrageenan	*Zymoseptoria tritici*	*Triticum aestivum*	Triggered SA- and JA-dependent signalling	Le Mire et al. (2019)

*SA salicylic acid, *JA* jasmonic acid, *ET* ethylene, *LOX* lipoxygenase, *ACO* ACC oxidase, *PR1, 2, 3, 5* Pathogenesis-related protein 1, 2, 3, 5, *AOS* allene oxide synthase, *CAT* catalase, *GPX* guaiacol peroxidase, *NPR1 nonexpressor of pathogenesis-related genes 1*, *PAL* phenylalanine ammonia-lyase, *PDF1*.2/*Def1.2*, *plant defensin*, *POD* peroxidase, *PPC* phenylpropanoid, *PPO* polyphenol oxidase, *SOD* superoxide dismutase

In addition to its direct antimicrobial effect, carrageenan pretreatment induces biochemical changes in host plants that result in tissue resistance to pathogen infection. In potatoes, carrots and chickpeas, carrageenan pretreatment has been shown to induce maximal browning and a high level of secondary metabolites (pathogen resistance chemicals) (Bi et al. 2008), suggesting that carrageenans are effective elicitors of plant resistance to pathogens. Production of carrageenan-induced secondary metabolites may help the plant defend itself from biotic and abiotic adversaries. Plants containing a high concentration of the induced secondary metabolites are thought to be more resistant to biotic and abiotic stressors (Zaynab et al. 2018). Moreover, carrageenan treatment can triggers the increased transcription of genes encoding pathogenesis-related proteins (PR proteins) (Vera et al. 2011; Mercier et al. 2001; Sangha et al. 2010). PR proteins are of great importance in inducing resistance against pathogens because of their potent antifungal and other antimicrobial action. To date, two types of induced resistance have been defined based on the pathways and defense genes involved (Hossain et al. 2008). Systemic acquired resistance (SAR) results from activation of salicylic acid (SA) pathways while induced systemic resistance (ISR) operates through jasmonate (JA), and ethylene (ET) pathways (Van Wees et al. 2000). Depending on the signaling chemicals, a particular set of PR proteins is stimulated (Heldt and Piechulla 2021; Hossain et al. 2008). The early-day research showed that depolymerized carrageenan kappa stimulated the PR protein activity, such as β-1,3 glucanase in *Rubus fruticosus* protoplasts and cells (Patier et al. 1995). Thus, carrageenans encouraged the accumulation of antimicrobial substances, which at least partly play a role in increased protection against plant diseases.

Tobacco leaves infiltrated with λ-carrageenan exhibited an enhancement in SA content, expression of JA and ET-inducible defense genes lipoxygenase (LOX) and ACC oxidase (ACO), and transcript levels encoding a chitinase, sesquiterpene cyclase, and proteinase inhibitor (Mercier et al. 2001). This study indicates that λ-carrageenan can modulate a diverse range of plant defense reactions, probably due to its high sulfate content. Sangha et al. (Sangha et al. 2010) demonstrated that λ-carrageenan induced resistance to *S. sclerotiorum* infection in *A. thaliana*, predominantly through a JA-dependent mechanism involving the transcription of the *PR3*, *PDF1*.2, and *allene oxide synthase* (*AOS*) defense genes. Vera et al. (Vera et al. 2011) stated that seaweed polysaccharides and oligosaccharides induce a local oxidative burst and systemic stimulation of SA, JA, and/or ET signaling pathways, leading to an enhanced transcription of genes encoding: (i) PR proteins, (ii) defense enzymes, including phenylalanine ammonia-lyase (PAL) and lipoxygense (LOX), and (iii) enzymes involved in the synthesis of terpenes. These enzymes are well-known for their antimicrobial activities. Later, Vera et al. (Vera et al. 2012) demonstrated that oligo-carrageenan treatment of tobacco modulates a sustained stimulation of PAL activity and the differential accumulation of several phenylpropanoid compounds with putative antimicrobial properties, implying that JA-mediated defense plays a major role in protection and suppression against TMV, *B. cinerea* and *P. carotovorum* in carrageenan-treated plants. Involvement of the JA pathway was also reported in tomato plants against TCDVD infection. λ-carrageenan treated tomato plants showed an increased function of LOX, AOS, and PR proteins, suggesting that the JA response may play a role in λ-carrageenan-mediated plant resistance to TCDVd infection (Sangha et al. 2015). Differences in protein expression patterns further imply the induction of biochemical changes in carrageenans-treated plants. A proteomic study of λ-carrageenan-treated Arabidopsis leaves followed by TCDVd infection revealed distinct variations in the abundance of proteins with various cellular activities (Sangha et al. 2015). Thus, carrageenan-induced metabolic changes in plants predominantly cause a reduction in viroid infection in carrageenan-treated plants. In a field trial, radiation-degraded carrageenan can induce resistance to tungro virus (Abad et al. 2018a). There was quite a high population density of beneficial arthropods in the carrageenan-treated field. These beneficial insects could control the green leafhoppers, which are the carriers of the tungro virus. This shows that in addition to inducing resistance induction, carrageenan supports the population of beneficial insects and controls viruses.

Furthermore, carrageenans can improve plant tolerance to stress by boosting the plant antioxidant machinery, which is primarily reliant on ROS-scavenging activity at the local and systemic levels. ROS are harmful byproducts of stress, such as plant diseases (Fürst et al. 2016). Plants possess antioxidant enzymes, such as CAT, POD, guaiacol peroxidase (GPX), ascorbate peroxidase (APX), polyphenol oxidase (PPO), and superoxide dismutase (SOD) to safeguard cells from oxidative injury and regulate the quantities of ROS. Plant tolerance may be correlated with a plant’s ability to scavenge ROS and lessen its harmful effects (Gill and Tuteja 2010). In response to the invasion by a particular pathogen, a carrageenan-induced plant may activate a unique set of responses, including triggering signaling pathways, defense genes encoding PR proteins, and the levels of antioxidant batteries. In the case of sweet basil, carrageenan treatment led to a significantly higher level of H_2_O_2_ and increased activities of CAT, PAL, and SOD while decreasing malondialdehyde (MDA), another aldehyde, and activities of PPO, LOX, or POD (Mousavi et al. 2018). In this study, the treatment of dodder-infected and non-infected basil plants with carrageenan might induce H_2_O_2_ production due to the transitory generation of ROS. Since low concentrations of H_2_O_2_ function as a secondary messenger to boost antioxidant activity, carrageenan is believed to induce the redox signal (H_2_O_2_ as) and increase enzyme activities (Mousavi et al. 2018). Therefore, carrageenan reduces oxidative stress injury in *Cuscuta*-infected plants by eliminating excessive ROS, preventing the JA-inducible LOX action and lipid peroxidation, and activating the enzymatic defenses like CAT, PAL, SOD, PPO, and POD (Mousavi et al. 2018). Applying κ-carrageenan to chilli leaves stimulated the production of the antioxidant enzyme POD, which is involved in plant defense against chilli anthracnose *Colletotrichum gloeosporioides* (Mani and Nagarathnam 2018).

Proteomic analyses of κ-carrageenan-treated chilli leaves also revealed upregulation various proteins, including NAD(P)H quinone oxidoreductase, dehydroascorbate reductase I, Eukaryotic Translation Initiation Factor 5A and dehydroascorbate reductase II (Mani and Nagarathnam 2018). NAD(P)H quinone oxidoreductase is reported to regulate PPO in plants via quinone and perform a crucial role in the maze defense against fungal infections (Wu et al. 2013). Dehydroascorbate reductase protects plants by keeping ascorbate in its reduced form (Eltayeb et al. 2006) and participates in incompatible interactions with pathogens (Kumar et al. 2015). Activation of Eukaryotic Translation Initiation Factor 5A has been reported during plant-pathogen interactions, particularly plant-virus contacts (Hopkins et al. 2008). Mani and Nagarathnam (2018) also identified a number of differentially expressed proteins associated with nitric oxide (NO) generation, PR protein expression, and phytoalexin synthesis, which include genes for encoding *CDKs*, *PR1*, and *NHO1*. The expression of *PR1* and *NHO1* indicates that both JA and SA are involved in chilli defense responses similar to their functions in Arabidopsis and wheat defense (Ding et al. 2011; Ishiga and Ichinose 2016). The gene expression study also supports this observation. Foliar applications of κ-carrageenan induced SA-inducible *PR1*, *PR5*, and *NPR1* genes and JA-inducible *PDF1*.2 genes in chili leaves, priming defense responses of both signaling pathways against anthracnose disease (*Colletotrichum gloeosporioides*) (Mani and Nagarathnam 2018). Expression of *PR1, PR2* and *PR5* has been documented during the enhanced defensive state conferred by pathogen-induced SAR (Kariola et al. 2003). In a study of SPS4-induced antiviral activity against TMV, the expression of the SA-dependent defense-related genes *PR1a* (Pathogenesis-related protein 1), *PR2* (β, 1, 3-glucanase), and *PR5* (thaumatin-like protein) and JA-regulated genes *PR3* (a basic chitinase) and *Def1*.2 (a defensin) was upregulated (Ghannam et al. 2013). Similarly, in semi-controlled conditions, foliar application of λ-carrageenan triggered both SA- and JA-dependent signaling pathways in wheat, protecting the plant from *Z. tritici* (Le Mire et al. 2019). Likewise, plant biostimulant oligochitosan can protect plants against fungi, bacteria, and viruses by activating the SA and JA–ET pathways (Moenne and González 2021). Application of fungal elicitor chitosan induced key genes in both SA (Xing et al. 2009) and JA (Farmer and Ryan 1992). In one study, alginate significantly upregulated the expression of SA-inducible *PR2*, and *NPR1*(nonexpressor of pathogenesis-related protein 1), ET-inducible *ACO1* (1-aminocyclopropane-1-carboxylate oxidase), and JA-inducible *LoxD* (lipoxygenase D) (Dey et al. 2019). Therefore, plant defense induced by carrageenans overlaps those reported in other plant biostimulants, such as chitosan and alginate. The involvement of both SAR (SA) and ISR (JA/ET) pathways in plant biostimulant-mediated defense responses suggests the contribution of multiple defense mechanisms. The interaction between defense pathways is intricate and has likely evolved to finely tune the plant defense mechanisms in response to the ever-changing pressure exerted by pathogens. Even though many studies have generally shown that SA and JA signaling pathways are antagonistic to one another, synergistic interactions have also previously been identified (Van Wees et al. 2000; Heldt and Piechulla 2021). The involvement of the SA pathway alone in carrageenan-mediated defense is not known much. However, Pettongkhao et al. (Pettongkhao et al. 2019) reported that the extracted crude polysaccharide from *Acanthophora spicifera* containing λ-carrageenan improves rubber tree defenses against *Phytophthora palmivora* by increasing the SA and scopoletin accumulation and SA-responsive gene expression while suppressing the JA-responsive gene expression. Besides, the purified λ-carrageenan induces POD activity but suppresses the CAT activity in tobacco leaves (Pettongkhao et al. 2019). These results demonstrate that carrageenans also promote SA-inducible defense.

**Fig. 5:**
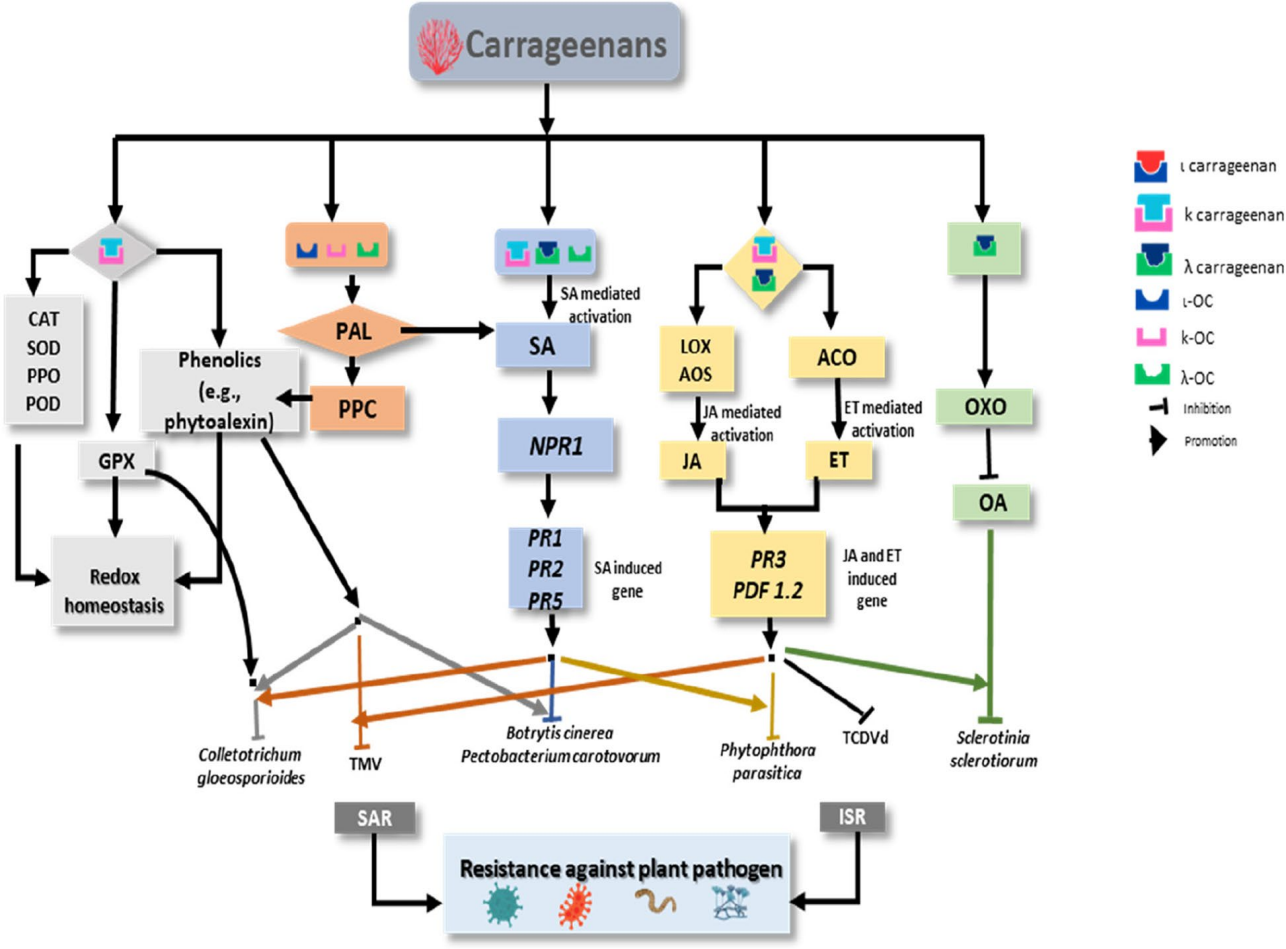
Molecular and biochemical mechanisms involved in plant defense against various diseases by carrageenans. Biochemical changes, signaling pathways and defense gene expression induced by carrageenan application in plants are indicated. ACO, ACC oxidase; AOS, allene oxide synthase; CAT, catalase; ET, ethylene; GPX, guaiacol peroxidase; ISR, induced systematic resistance; SA, salicylic acid; JA, jasmonic acid; LOX, lipoxygenase; *PR1, 2, 3, 5*, *Pathogenesis related protein 1, 2, 3, 5*; *NPR1*, *non-expressor of pathogenesis-related genes 1*; *PDF1*.2, *plant defensin*; OA, oxalic acid; OXO, oxalate oxidase; PAL, phenylalanine ammonia-lyase; POD, peroxidase; PPC, phenylpropanoid; PPO, polyphenol oxidase; SAR, systemic acquired resistance; SOD, superoxide dismutase

Similar to carrageenans, pretreatment of Arabidopsis with pectin oligosaccharides triggered robust defense responses mediated by the SA signaling pathway, resulting in resistance against *Pseudomonas syringae* Pst DC3000 (Howlader et al. 2020). Equally, alginate oligosaccharides also induced defense responses through SA-mediated signaling pathways (Zhang et al. 2019). Consequently, λ-carrageenan exhibits a resemblance in its reliance on the SA signaling pathways to induce defense responses, analogous to the mechanisms of alginate and pectin oligosaccharides. In contrast, ulvan induces the expression of JA-dependent genes, such as *PDF1.2* defensin in *Arabidopsis thaliana* and the lipoxygenase NtLOX1 promoter in *Nicotiana tabacum* (Penninckx et al. 1996; Fammartino et al. 2007). In a study with *Medicago truncatula*, ulvan also triggered a gene expression signature similar to that observed with methyl jasmonate (MeJA) (Jaulneau et al. 2010). Consequently, ulvan appears to exhibit distinct inducible defense mechanisms from λ-carrageenan in *N. tabacum* and *A. thaliana*. Various forms of induced resistance exist, and their reliance on plant regulators may vary depending on the plant, pathogen and biostimulant interaction. Furthermore, these signaling pathways are not straightforward, linear, or isolated cascades; they often cross-talk. The versatile plant defense mechanisms induced by carrageenans against various pathogens are summarized in Fig. 5.

## Conclusions

Application of seaweed carrageenans as biostimulants and bio-elicitors has significant potential for enhancing plant growth and defense systems against biotic stresses in the backdrop of climate change. Identification of the complex interaction between these chemicals and the physiological processes of plants is essential to address the negative impacts of changing environmental circumstances. Carrageenans can contribute substantially to sustainable agriculture by improving plant growth, stress tolerance, and overall resilience. These potential holds promise for developing more resilient and adaptable plant systems in a progressively challenging climatic scenario. However, challenges, constraints and possible solutions in commercial carrageenan production and application must be identified for the industry’s future growth. Developing novel production techniques, optimizing the existing methods, and the genetic selection of high-yielding seaweed species can improve the carrageenan yield extraction and reduce the cost of production necessary for broader adaptation by the farmers. Genetic engineering can be employed to develop genetically modified algal strains that can produce high commercial-value carrageenan. Further research into degrading the naturally occurring high molecular weight carrageenan into low molecular weight smaller oligomers can increase the effectiveness and stability of the elicitor in plants.

Although enormous progress has been made over the past two decades in understanding the mechanisms of carrageenans implicated in stimulating growth and defense against pathogens, additional inclusive investigation can identify green plant target molecules interacting with carrageenans, revealing novel regulatory relationships affecting plant development and defense systems. A deeper understanding of the regulatory relationships can have implications in biotechnology and bioengineering by providing insights into potential crop improvement and biostimulation applications. The advancement of the current understanding regarding the suitable formulation and optimization for storage, transportation, and application is crucial for the success of carrageenan-based natural products as plant growth promoters and defense elicitors. Conducting large-scale field-based studies is imperative to confirm the impact of these formulated products on crops. Ultimately, a strong linkage between academia and industries could pave the way for developing a green, multifunctional bio-fertilizer to boost crop yields and ensure agricultural sustainability amidst the global climate crisis.

## Abbreviations

ACO: ACC oxidase
AOS: Allene oxide synthase
CAT: Catalase
ET: Ethylene
GPX: Guaiacol peroxidase
ISR: Induced systematic resistance
SA: Salicylic acid
JA: Jasmonic acid
LOX: Lipoxygenase
OA: Oxalic acid
OXO: Oxalate oxidase
PAL: Phenylalanine ammonia-lyase
POD: Peroxidase
PPC: Phenylpropanoid
PPO: Polyphenol oxidase
PRX: Class III peroxidase
ROS: Reactive oxygen species
SAMS: S-adenosylmethionine synthase
SAR: Systemic acquired resistance
SOD: Superoxide dismutase
TCM: Trans-cinnamate 4-monooxygenase
